# Supplementary material for: Neural and behavioral adaptations to frontal theta neurofeedback training: A proof of concept study
Source: PLoS One. 2023 Mar 23;18(3):e0283418. doi: 10.1371/journal.pone.0283418 (PMC10035884; doi:10.1371/journal.pone.0283418)
Supplement: S1 Fig — (DOCX) [file pone.0283418.s001.docx]

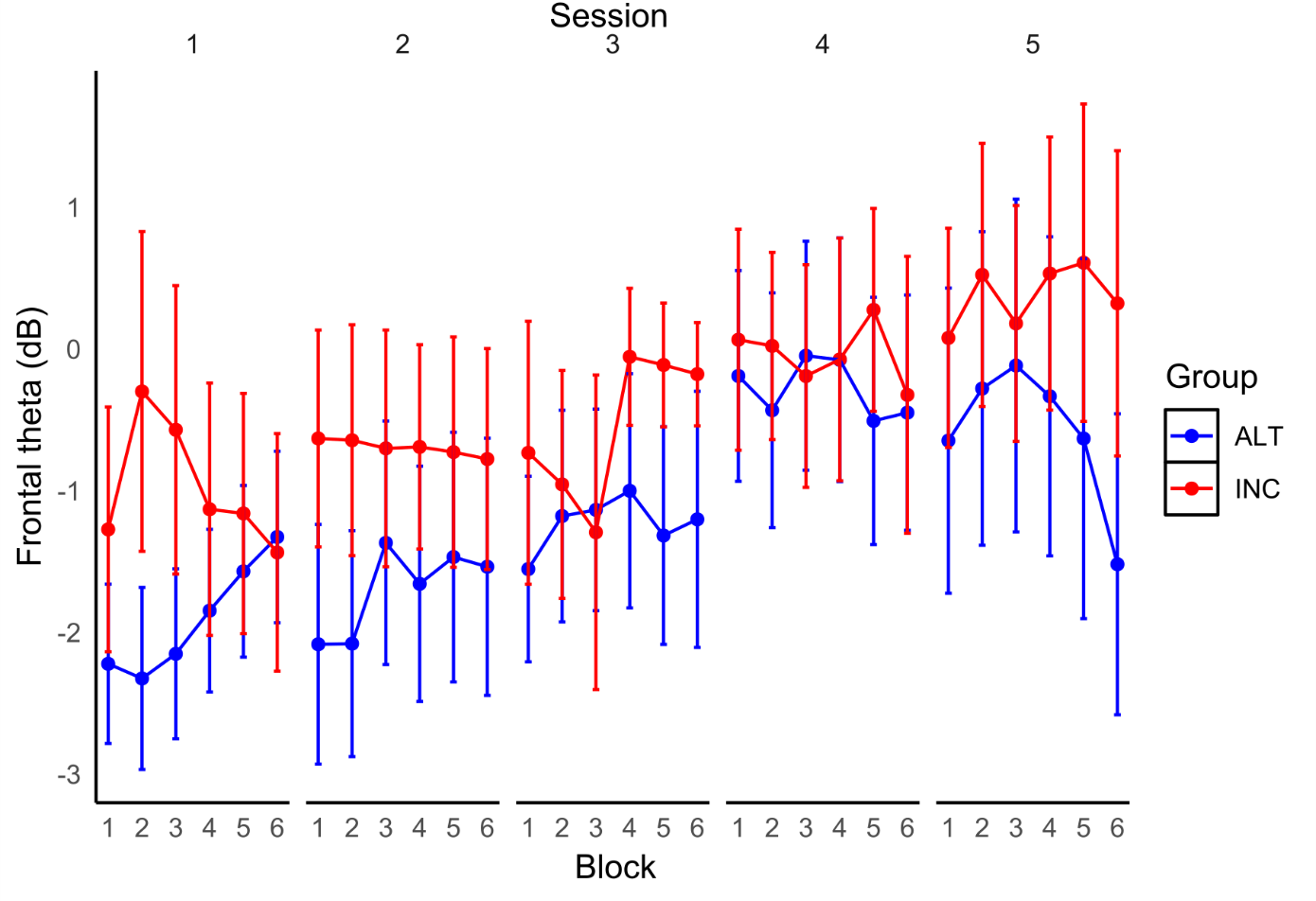


**S1A Fig. Changes in Fmθ over blocks and sessions of NF training for each Group in Responders-Only analysis.** Changes in frontal theta power over blocks and sessions of NF training for each Group using responders-only (error bars are SE).


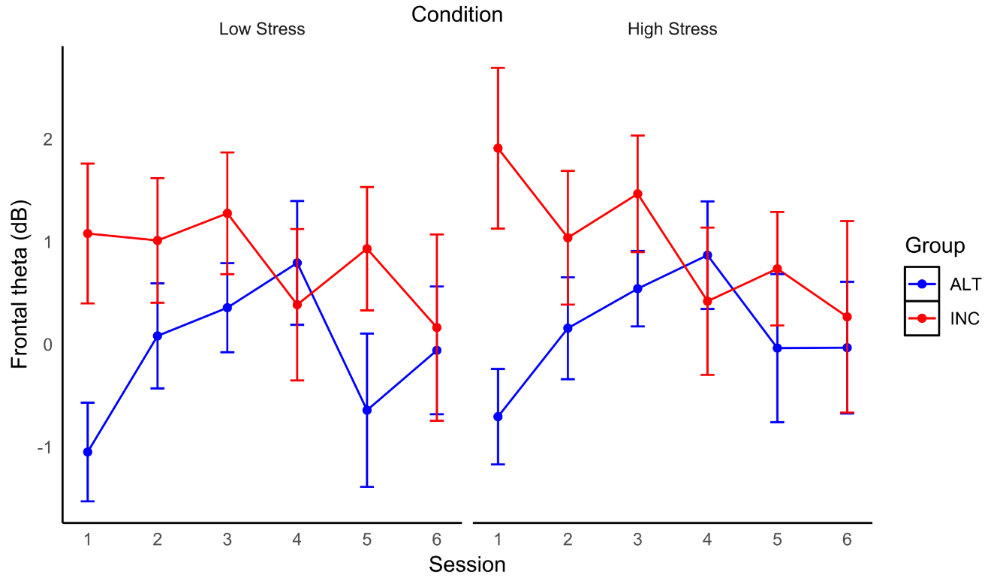


**S1B Fig. Fmθ changes across Sessions in Low and High time-stress conditions for each Group during SH in Responders-Only analysis.** Changes in Fmθ over Sessions during Go-NoGo shooting task in Low (left) and High (right) time-stress conditions for each Group using responders-only (error bars are SE).


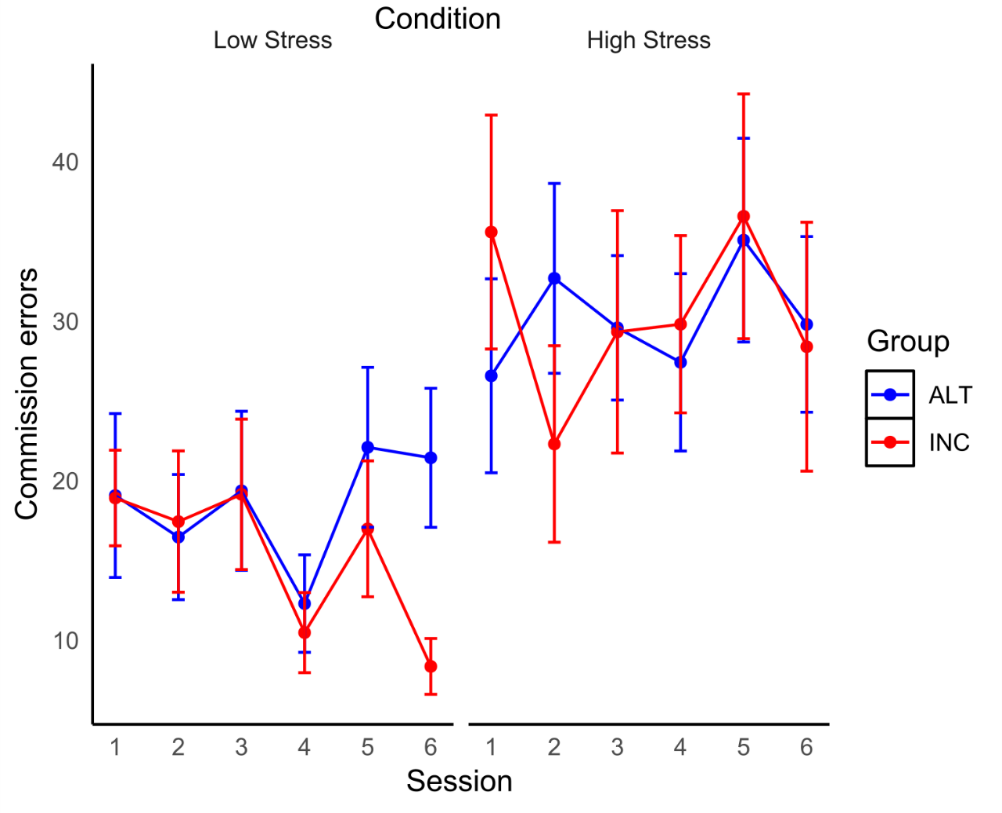


**S1C Fig. Changes in errors of commission across Sessions in Low and High time-stress conditions for each Group in Responders-Only analysis.** Percentage errors of commission over Sessions in the Low (left) and High (right) time-stress conditions for each Group using responders-only (error bars are SE).


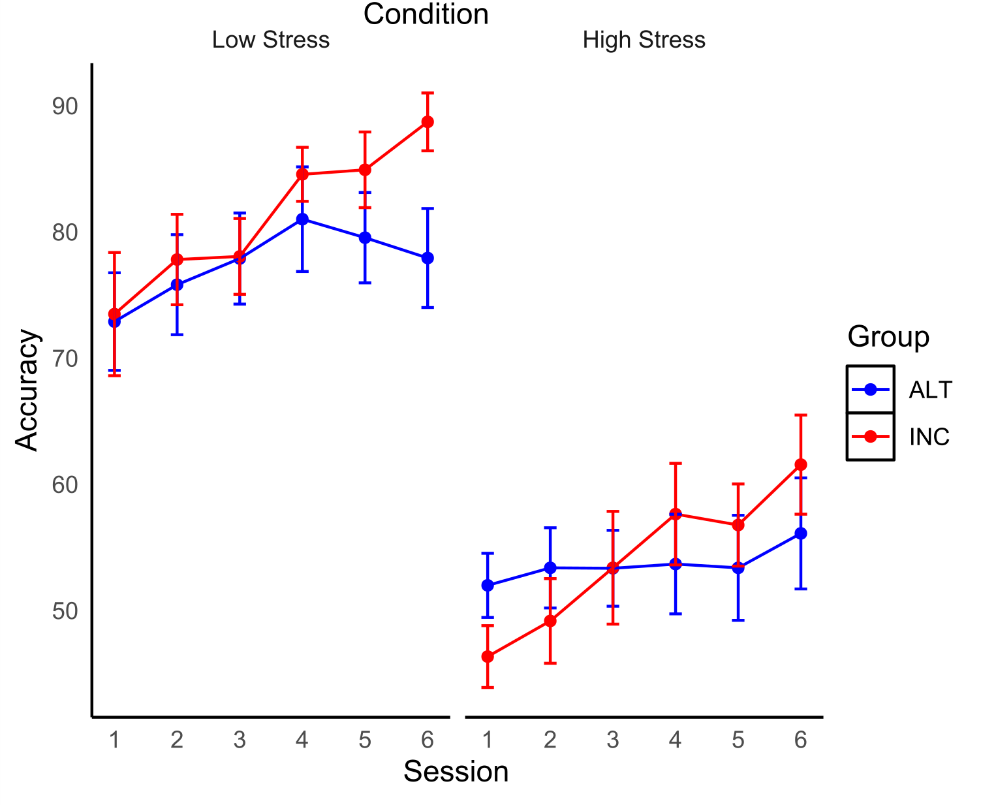


**S1D Fig. Changes in shooting accuracy across Sessions in Low and High time-stress conditions for each Group in Responders-Only analysis.** Percentage of enemy targets hit in the Low (left) and High (right) time-stress conditions by session for each group using responders-only.


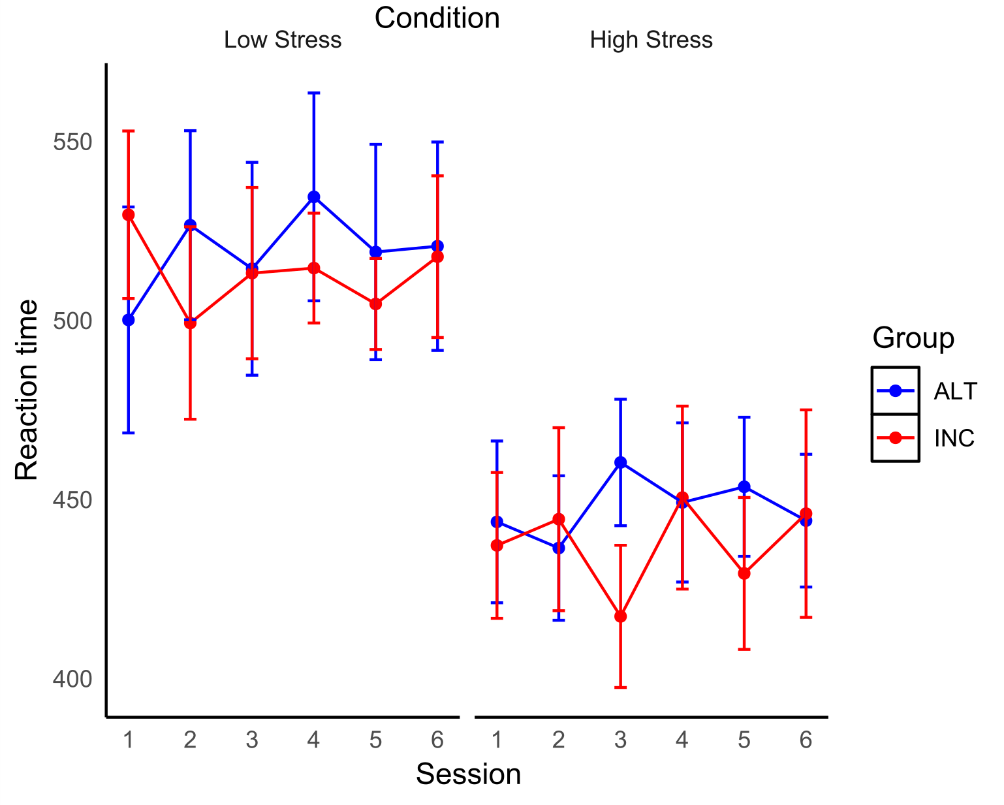


**S1E Fig. Changes in RTs across Sessions in Low and High time-stress conditions for each Group in Responders-Only analysis.** RTs to enemy targets in the Low (left) and High (right) time-stress conditions by session for each group using responders-only.
