# Supplementary material for: Neural and behavioral adaptations to frontal theta neurofeedback training: A proof of concept study
Source: PLoS One. 2023 Mar 23;18(3):e0283418. doi: 10.1371/journal.pone.0283418 (PMC10035884; doi:10.1371/journal.pone.0283418)
Supplement: S1 Table — (DOCX) [file pone.0283418.s002.docx]

**S1A Table. Full MLM of Fmθ during NF training Responders-Only**

|  | Frontal theta |  |
| --- | --- | --- |
|  | β | *p* |
| (Intercept) | -0.24 (0.97) | 0.81 |
| Modulation | 0.02 (0.12) | 0.85 |
| Block | -0.13 (0.1) | 0.19 |
| Session | -0.21 (0.33) | 0.55 |
| Group | -3.36 (1.23) | **0.01** |
| Block*Session | 0.03 (0.03) | 0.28 |
| Group*Block | 0.41 (0.12) | **< .01** |
| Group*Session | 0.99 (0.43) | **0.03** |
| Group*Session* Block | -0.11 (0.04) | **< .01** |
| Variance for session slope | 0.82 |  |
| ICC | .84 |  |
| Fixed R^2^ | .04 |  |
| AIC | 1618.81 |  |
| BIC | 1673.60 |  |

**S1B Table. Full MLMs of Fmθ during Go-NoGo shooting task Responders-Only**

|  | Frontal theta |  |
| --- | --- | --- |
|  | β | *p* |
| (Intercept) | 1.27 (0.80) | .13 |
| Condition | 0.22 (0.44) | .62 |
| Session | -0.13 (0.19) | .50 |
| Group | -1.57 (1.03) | .14 |
| Condition*Session | -0.02 (0.11) | .89 |
| Group*Condition | 0.00 (0.54) | 1.00 |
| Group* Session | 0.23 (0.24) | .36 |
| Group*Condition*Session | 0.00 (0.14) | 1.00 |
| Variance for session slope | 0.46 |  |
| ICC | .76 |  |
| Fixed R^2^ | .03 |  |
| AIC | 1383.43 |  |
| BIC | 1431.45 |  |

**S1C Table. Full MLMs for mehavioral measures from Go-NoGo shooting task Responders-Only**

|  | Commission errors |  | Accuracy enemies |  | Reaction time enemies |  |
| --- | --- | --- | --- | --- | --- | --- |
|  | β | *p* | β | *p* | β | *p* |
| (Intercept) | 20.85 (5.92) | **< .01** | 71.16 (4.4) | **< .01** | 534.65 (27.97) | **< .01** |
| Condition | 9.71 (5.88) | .10 | -28.58 (3.37) | **< .01** | -104.09 (17.56) | **< .01** |
| Session | -1.56 (1.10) | .16 | 2.84 (0.97) | **.01** | -3.33 (4.18) | .43 |
| Group | -4.73 (7.57) | .54 | 2.02 (5.65) | .72 | -11.11 (35.71) | .76 |
| Condition*Session | 1.73 (1.51) | .25 | 0.36 (0.88) | .68 | 4.52 (4.5) | .32 |
| Group*Condition | 3.89 (7.55) | .61 | 3.44 (4.34) | .43 | 25.38 (22.24) | .26 |
| Group*Session | 2.13 (1.40) | .14 | -1.63 (1.24) | .20 | 3.56 (5.3) | .51 |
| Group*Session*Condition | -1.93 (1.93) | .32 | -0.31 (1.12) | .78 | -5.5 (5.68) | .33 |
| Variance for session slope | 0.84 |  | 1.99 |  | 6.97 |  |
| ICC | .51 |  | .70 |  | .81 |  |
| Fixed R^2^ | .18 |  | .59 |  | .25 |  |
| AIC | 1554.35 |  | 1434.42 |  | 1993.07 |  |
| BIC | 1593.81 |  | 1474.35 |  | 2032.53 |  |
