# Supplementary material for: Neural and behavioral adaptations to frontal theta neurofeedback training: A proof of concept study
Source: PLoS One. 2023 Mar 23;18(3):e0283418. doi: 10.1371/journal.pone.0283418 (PMC10035884; doi:10.1371/journal.pone.0283418)
Supplement: S1 Text — (DOCX) [file pone.0283418.s003.docx]

## Neurofeedback Training Responders Only

An analysis using the full model was conducted to examine Fmθ using only participants that were considered responder. Similar to using the full dataset it was found that there were statistically significant interactions of Group by Block (b = 0.41, p < .01), Group by Session (b = 0.99, p = .03), and Group by Session by Block (b = -0.11, p < .01). One difference that was observed in the responders-only analysis was that there was a statistically significant effect of Group (b = -3.36, p = .01) indicating that participants in the ALT group had a lower average Fmθ.

## Go-NoGo Shooting Task Responders-Only

An analysis was also conducted of Fmθ from shooting task trials using the full model on responders-only. There were no differences between the model using the full dataset and responders-only. Predictors were not statistically significantly related to Fmθ during the shooting task.

### Behavioral Performance Responders-Only

#### % Errors of Commission

Several differences were observed between the analysis using the full dataset and responders-only. Condition and Session were no longer statistically significant predictors of Commission errors.

#### Accuracy

When using responders-only Condition (b = -28.58, p < .01) and Session (b = 2.84, p < .01) remained statistically significant predictors of Accuracy. However, the Group by Session interaction was no longer statistically significant.

#### Reaction Times

Finally, an analysis of reaction times using responders-only led to the effect of Condition remaining statistically significant (b = -104.09, p < .01), but Condition by Session and Group by Condition interactions were no longer statistically significant.
