# Supplementary material for: Neural and behavioral adaptations to frontal theta neurofeedback training: A proof of concept study
Source: PLoS One. 2023 Mar 23;18(3):e0283418. doi: 10.1371/journal.pone.0283418 (PMC10035884; doi:10.1371/journal.pone.0283418)
Supplement: Attachment — Submitted filename: Responses to Reviewers.docx [file pone.0283418.s004.docx]

```
      View Letter

Date:	Jan 10 2022 10:25AM
To:	"Scott E. Kerick" scott.e.kerick.civ@mail.mil
From:	"PLOS ONE" plosone@plos.org
Subject:	PLOS ONE Decision: Revision required [PONE-D-21-31557]

PONE-D-21-31557
Neural and behavioral adaptations to frontal theta neurofeedback training

PLOS ONE

Dear Dr. Kerick,

Thank you for submitting your manuscript to PLOS ONE. After careful
consideration, we feel that it has merit but does not fully meet PLOS
ONE’s publication criteria as it currently stands. Therefore, we invite
you to submit a revised version of the manuscript that addresses the
points raised during the review process.

1) In lines 54-55 authors postulated that neurofeedback (NF) "is a
highly attention-demanding task that must be learned through extended
deliberate practice like many skills." This is not in accordance with
the fact that in their experiment they only give 5 sessions. This should
be discussed. Even in operant conditioning learning that compromises
survival, such a small number of sessions does not make learning happen;
It is difficult then to believe that it happens when life is not
threatened ...

We agree that the extent of learning afforded in five sessions does not constitute extended deliberate practice. The statement was in fact intended to make this point, that learning to control specific features of one’s brain recordings in a limited number of sessions does not constitute mastery or advanced learning. We moved this statement in the Introduction to the Discussion under “Limitations and Future Directions”.

2) lines 82-100: Up-regulation of Fmθ has been used to improve the
cognitive performance in healthy young subjects. Up-regulation of Fmθ
protocol should not be confused with the protocol to promote REDUCTION
in theta power, used by Becerra et al. (2012), in which the subjects
were older adults at risk of cognitive decline (evidenced by an excess
of theta power for their age, when compared with normative values).
Please review the rationale of this last paper.
In this study, a lead was selected in each subject to give the NF.
Although in 7 of 14 subjects a frontal lead was selected (in 3 from the
experimental group and in 4 from the control group), Fz was never
selected; i.e., no subject was down-regulated Fmθ. Therefore, the
sentence in lines 98-100 ("Taken together, there is preliminary evidence
that either up regulating or down-regulating Fmθ can potentially promote
gains in executive performance of some tasks) is not true.
A crucial point in this regard is that these older adults had excess
theta at rest, and must be undergoing some compensatory process to
correct this abnormality, since their cognitive performance is normal;
while the Fmθ protocol of the other studies mentioned has been applied
mainly to young subjects for whom the normality / abnormality of their
EEG is unknown.

We agree with this important criticism. Becerra et al., and much of the literature on Fmθ NF training, has been done on patient populations (e.g., ADHD, elderly) which exhibit excess frontal theta thought to be disruptive to cognitive performance. We revised our referencing of this study by more detailed review of this and other NF studies originally cited, stating that Fmθ was not modulated in Becerra et al., and describing the differences between studies on healthy young adults and elderly/patient. We then focus on the fact that no previous researchers have considered alternating Fmθ in a manner that is more consistent with frontal theta dynamics (increases/decreases) during executive control tasks (activation/inhibition); see revised section “Theta: A promising neural target for training executive control”.

3) lines 105-108: Considering that accurate contingent feedback linking
response and reward is required for optimal learning to occur, it’s
difficult to disentangle whether NF training-related differences are due
to reinforcement learning based on contingent reward mechanisms or
increased/decreased theta power levels per se, or both.
I consider that this paragraph forms the cornerstone of this study.
Consequently, it must be very clearly formulated. In previous studies,
two groups have been considered: an experimental group, in which Fmθ has
been up-regulated, and a control sham group.  It is difficult to
consider separately the theta increase at Fz lead and the accuracy of
contingent feedback. Therefore I ask the authors to take in
consideration the following facts to rephrase their research question:
     • In the experimental group, there are:
             o increased  θ power at Fz
             o accurate contingent feedback to ensure optimal learning
     • In the control group,:
             o no optimal learning can be ensured because there is not an accurate contingent feedback
If authors consider that the previous facts are incorrect, please
explain why.
```

We agree with these comments and facts. Accurate contingent feedback is essential for optimal learning to occur. However, just because accurate contingent feedback is provided in NF training groups but not sham control groups, does not necessarily mean that greater increases in Fmθ in the NF group is clear and unambiguous evidence that it is because of this coupling between accurate contingent feedback and increased theta power. It cannot be assumed, alternative hypotheses should be rejected. The increased theta could alternatively be a function of different levels of motivation, attentional engagement, task difficulty, strategies, or any of a number of other possibilities. We have attempted to more clearly make this point in the revision under the section “Unresolved Issues and Gaps”.

4) lines 113-114: "Another key limitation of existing NF studies,

including those targeting Fmθ, is that they modulate neural activity

only in one direction (either solely up-modulation or solely

down-modulation)." The authors should explain why they do it; that is,

their rationale based on EEG activity during attention tasks (Gevins et

al., 1997).

The main point here is that, although higher levels of frontal theta characterize attentional engagement in attention tasks, it is not stationary. Rather, as event-related spectral perturbation studies indicate, theta is phasically modulated across trials of executive tasks. Therefore, it is tenable to conjecture that alternating the power of frontal theta over the course of training may be a more task-specific training protocol than exclusively increasing it to that which is exhibited during task performance (task specificity). An analogy might be training a sprinter (50 m) by having him/her run long distances (5 K).

In lines 76-83, under “Theta: A promising neural target for training executive control”, it was our intent to provide a rationale for why NF training is designed to increase Fmθ power:–

“Extensive research has shown that EEG-theta synchronization is central to executive control (Cavanagh et al., 2009; Luu et al., 2004; Mizuhara & Yamaguchi, 2007; Polania et al., 2012; Womelsdorf, Johnston, Vinck, & Everling, 2010; Womelsdorf, Vinck, Leung, & Everling, 2010). Theta synchronization functions to enable long-distance communication in the brain, dynamically linking subcortical and cortical brain networks associated with integrating and coordinating perceptual, cognitive, and motor control functions.”

However, our main point in lines 113-114 was to emphasize the dynamic and nonstationary nature of phasic theta changes during attention tasks:

“However, to support adaptive behavior in real-world situations, neural activity must exhibit dynamic variability such that activation and inhibition must be coordinated in response to varied internal/external demands.”

We agree that Gevins et al. (1997) is a relevant citation to include in making this point that frontal theta increases in response to executive task demands, and therefore provides further justification for up-modulating Fmθ in NF training. We have included this reference in the above paragraph stating that EEG-theta synchronization is central to executive control.

5) It must be made very clearly established that this doubt about the

placebo sham treatment is only applicable to the Fmθ protocol. However,

when using protocols other than this one, the argument that attention

could be the one that increased theta activity in Fz has no place.

We agree, that this issue is specific to Fmθ. We believe that we make this clear in the first few sentences under “Unresolved Issues and Gaps”

“Representing another key issue, analogous to the above on sham control issues, the Fmθ activation targeted by NF is often confounded with the neural functions required to modulate brain responses in NF protocols. Specifically, NF is an attention-demanding task requiring the activation of frontal executive control networks during training irrespective of the frequency or region targeted by feedback (Gruzelier, 2014; Haller et al., 2013; Emmert et al., 2016). Since Fmθ is implicated in such executive control processes, observed Fmθ increases during NF training may be erroneously attributed to effective NF training (i.e. reinforcement learning based on accurate contingent feedback) when such increases may merely indicate sustained deployment of attention and/or cognitive effort.”

6) I agree with Reviewer 2 that it is difficult to reach the conclusions

the authors reach from the results presented. Perhaps by assembling the

results in another way, the authors' conclusion could be accepted, but

it is not clear to me in the current form.

We are not sure how to reassemble the results in a more clear or refined way. We believe it is important to first show how the different training protocols resulted in different Fmθ changes between groups over blocks and sessions. Establishing that the ALT group exhibited higher levels of Fmθ over blocks and sessions suggests that accurate contingent reward cannot account for these findings. If feedback enabled learning to occur, then the ALT group should have been able to downregulate their Fmθ levels more during down vs up-regulation blocks, and they should have generated lower Fmθ than the INC group across both blocks and sessions. Therefore, we conclude that some other mechanisms must be accountable for this finding (e.g., differences in cognitive demands, fatigue, or other).

Then, to test how the training affected Fmθ and performance during the Go-NoGo task, we follow with neural and behavioral results. The findings are a bit difficult to clearly comprehend, as no differences were observed for Fmθ, while behavioral results differed depending on the particular measure. However, short of leaving out specific results for convenience or brevity, it’s hard to portray the complete picture. We hope that by revising our Introduction, the questions and hypotheses are more clear, thereby making the Results and Discussion/Conclusions more clear.

7) Not having solved this point (6), it is difficult to form an opinion

of the Discussion.

Please see reply to (6) above.

------------------------------------------------------------------------

5. Review Comments to the Author

Reviewer #1: In literature, there is no consensus of how to design aт

ultimate neurofeedback study: what EEG parameter to train, what

instruction to give, what a comparison neurofeedback option (SHAM?) to

use, and what behavioral/neuronal indexes apply to measure the changes.

We agree with this comment.

This study uses a good rational for selection a neurofeedback study: 1)

a frontal midline theta rhythm (4-7 Hz) measured at FCz selected as a

neurofeedback parameter. 2) No specific instruction was done to modulate

the parameter. 3) Reaction time in shooting task (SH) and EEG spectra

were used to measure pre-post differences. 4) Two groups with the two

types of NF procedures: a) steadily increase theta – INC, and b)

alternatively increase/decrease theta - ALT. The study required a lot of

hard work of skilled professionals.

However, looking at the results presented in Fig. 4-10, I can’t say that

the results are impressive. My impression is that no differences between

the groups are found. To prove the opposite (the one that the authors

claim in the text), the authors should try to assemble the results in a

different and condensed way.

Based on our statistical analyses, we did observe significant group differences in Fmθ over blocks and sessions of NF training, and behavioral measures in the Go-NoGo task, but we did not observe differences in Fmθ over sessions in the Go-NoGo task. These findings are difficult to clearly interpret, and may be considered unimpressive. However, we argue that our results contradict previous research, and that reporting these results is relevant to the corpus of NF literature in stimulating new research that should be designed to reject alternative hypotheses (rather than support existing ones) that training-related increases in Fmθ underlie executive task improvement. In our view, based on our results alternative hypotheses remain tenable, and creative implementation of alternative control groups beyond just sham control should be encouraged to advance our understanding of NF training.

Further, if we accept the results as positive (i.e. the study shows

changes between the groups in EEG spectra and behavioral parameters) the

Discussion is worth to read.

We hope that by revising our Introduction, the questions and hypotheses are more clear, thereby making the Results and Discussion/Conclusions more clear.

Reviewer #2: This study encompasses de comparison between two different

neurofeedback protocols (increase of Theta and increase/decrease Theta)

which aimed at assessing the specificity of NF training overcoming the

methodological difficulties of sham and placebo groups.

The authors did a great job, the methods are clear, and the analyses are

very good. Just in case, the authors might be interested in the Bayesian

LM (i.e. <https://pubmed.ncbi.nlm.nih.gov/33486138/>)

We thank the reviewer for the suggestion. We view Konicar et al., (2021) work to be an interesting approach to analyzing neurofeedback data. Given our data distribution we feel that our analyses (in this case) are best supported by using restricted maximum likelihood—as part of the multi-level modeling approach (maximum likelihood is a core component of Bayesian analyses).

Here my minor comments:

Intro:

L 93 The authors state that less NF research were down to down training

Theta band, however there is a large literature in ADHD which in fact

trained down-regulation, although mainly together with up-regulation of

the Beta band. This might be mentioned. Here a new reference

Arnold, L. Eugene, Martijn Arns, Justin Barterian, Rachel Bergman, Sarah

Black, C. Keith Conners, Shea Connor, u. a. „Double-Blind

Placebo-Controlled Randomized Clinical Trial of Neurofeedback for

Attention-Deficit/Hyperactivity Disorder With 13 Month Follow-Up“.

Journal of the American Academy of Child & Adolescent Psychiatry, 25.

August 2020. <https://doi.org/10.1016/j.jaac.2020.07.906>.

We thank the reviewer for the suggestion and reference. We have incorporated a few sentences acknowledging that downregulation of frontal theta is frequently implemented in research on elderly and patient groups and included this reference, among a few more.

Methods and discussion: Are clear and well written. However, I miss the

question regarding specificity and I would recommend performing an

additional analysis, which takes into account the block, and session

wise learning (i.e. blocke wise / session wise slope on Task

performance) and introduce this into the model of the No-go Task. As far

as I understood, this was not made. It would add important evidence (or

not) of specify of the training which is an important topic.

In general we agree with the reviewer. However, there are a few reasons why we did not analyze the data at the block level for the Go-NoGo task. First, the block-level data consists of only 9 no-go trials (10% of 90), making analyses of Fmθ to friendly targets and errors of commission unstable. Second, we are concerned about overfitting a model with additional factors (Group, Session, Condition, Block) given only 30 subjects. Third, for the first two reasons, we did not partition the data at the block level, and doing so now would take more time than we have to submit a revision.

Reviewer #3: The research question of this study is quite interesting.

However, the proposed methodology does not answer the question. The

major problem is that the hypotheses are false, i.e., they are presented

as mutually exclusive when, in fact, they are not.

I present some issues that the authors should consider:

INTRODUCTION

- There are several conceptual inaccuracies throughout the introduction.

The scope of NF training (i.e., the operant conditioning of brain

activity) should be kept in mind in order to avoid mereological

fallacies (e.g. lines 67 - 68: NF does NOT train executive control, it

trains changes in brain activity, which are not equivalent).

We agree with this important criticism. We have revised this sentence: “As such, using NF to train modulation of neural functions related to executive control”, and throughout the Introduction. We agree with Rogala et al. (2016), who claimed that “NFB protocols can be measured by unambiguous changes in EEG activity and by changes in the targeted cognitive function”. We have incorporated Rogala et al. in paragraph two under the sub-heading: “Theta: A promising neural target for training executive control”.

- The authors present a very poor review of previous research regarding

fmTheta NF. Only 3 previous studies are shown. More importantly, the 3

studies follow very different rationales:

We realize that there is a wealth of research on NF training among various subject populations (children/adult/elderly, clinical/normal) targeting various frequency bands, brain regions, and training goals. However, there is a paucity of research on Fmθ NF training on healthy young adults for cognitive performance enhancement. Many studies have investigated ratios of frontal theta with other frequency bands (e.g., alpha, beta); however, training-related changes of ratios are difficult to interpret because it is not known whether the changes observed are due to theta or some other band, or both, unless absolute values are also included. Further, elderly and clinical populations generally exhibit overactive frontal theta, which is why down-regulation goals of training are implemented, but these populations differ from our young healthy subjects and our training goals are different. For this reason, we necessarily limited our literature review to studies specifically designed to investigate purely Fmθ NF training on healthy young adults for cognitive performance enhancement, which required us to summarize studies with different rationales. We have incorporated more detailed review of the most highly relevant studies to better illuminate consistencies/inconsistencies, and outstanding issues. We based most of our review of relevant literature on a recent review by Rogala et al. (2016), which we have inserted in paragraph two under “Theta: A promising neural target for training executive control”:

In their recent meta-analytic review of the NF literature, Rogala et al. (2016) concluded that, “the validity of EEG-NFB protocols can be measured by unambiguous changes in EEG activity and by changes in the targeted cognitive function. Unfortunately, most of the work conducted in the EEG-NFB field has failed to satisfy the unambiguity criterion for both of the variables and the field itself has shown a big tolerance for violations of scientific methodology” (Rogala et al., 2016, p. 2). They went on to say that “a rigorous scientific approach to EEG-NFB is rare, and **experiments performed on** **healthy participants to study the effectiveness and/or mechanism of training are very limited**” (Rogala et al., 2016, p. 8). Based on their review, **only five theta NF studies met these criteria** (Becerra et al., 2011; Enriquez-Geppert et al., 2013; Enriquez-Geppert et al., 2014; Reis et al., 2015; Wang & Hsieh et al., 2013). Four of five studies implemented theta up-regulation, and all five revsealed significant EEG and behavioral changes in NF vs. control groups (more recently, we also included Brandmeyer & Delorme, 2020).

1. Enriquez-Geppert et al., 2014. They sought to increase fmTheta using

NF based on task-induced theta, which is very different to

resting-state, task-independent theta activity.

Enriquez-Geppert et al. (2014) did use executive control tasks to determine individual peak theta frequencies for training; however, the threshold levels of resting baseline Fmθ preceding NF training was used as reference thresholds for each training session: “the raw power value of each 2 s segment for the individualized fm-theta frequency was compared to the baseline power of the same frequency… power change [was] relative to the start-baseline of the specific NF session” (p. 5). Further the other studies we reviewed also used pre-training rest baselines as threshold for training (Brandmeyer & Delorme, 2020; Wang & Hsieh, 2013). We adopted this approach in our study in the first session (training threshold based on preceding resting state Fmθ), but after the first session, in subsequent sessions, we used the mean Fmθ from the preceding session as the threshold. In this way, our approach was actually based on Fmθ generated during NF training sessions, after the first session. We believe this approach would make training more task-dependent. Further, we know from previous research that frontal theta phasically increases in response to targets and non-targets in Go-NoGo shooting tasks (Kerick, Hatfield, & Allender, 2007; <https://www.ingentaconnect.com/content/asma/asem/2007/00000078/A00105s1/art00024>), so our training was not based on resting state theta.

2. Brandmeyer & Delorme, 2020. They also sought to increase fmTheta

using NF based on the characteristics of task-specific brain activity,

that is, during meditation.

Brandmeyer & Delorme (2020) did have their subjects modulate Fmθ while simultaneously monitoring breathing cycles, which actually was a form of dual-task training. This does complicate the interpretation to some extent. However, given the relative paucity of Fmθ neurofeedback research on healthy individuals designed to improve executive function, we feel it is important to include this study in our summary of the relevant literature. To point out the different characteristics of this study, we have elaborated our review of it in the Introduction. As mentioned above, Brandmeyer & Delorme also based their Fmθ training thresholds on resting state theta preceding training in the first block if the first session, and subsequently the threshold was based on Fmθ from the previous block: “… the 1 min preparatory baseline period which preceded the first feedback session which allowed the program to calculate an acceptable theta dynamic range for the onset of the first neurofeedback block. Dynamic range at the end of each of the 5 min block, was used as a starting point for the following 5 min Blocks” (p. 6). Our thresholds for training were implemented similar to this: thresholds were based on resting theta in first session, but then was based on changes over training sessions.

3. Becerra et al., 2012. The description of this article in the

introduction is mistaken. Moreover, the rationale behind the NFB

treatment has nothing to do with the task-specific fmTheta activity.

We agree that the description of Becerra et al. was not accurately portrayed and should not have been discussed in the context of decreasing Fmθ in healthy young adults. In that study, elderly subjects with abnormally high levels of theta across various brain regions were tested. Accordingly, we have revised the manuscript under the sub-heading “Theta: A promising neural target for training executive control”.

In general, we argue that NF may be task-specific or general, depending on the goals of the training, targeted neural features, and training schedule (Papo, 2019). Specific to Becerra et al., we agree that training was not designed with task-specificity in mind: “The goal of this work was to explore the effectiveness of a NFB protocol in reducing theta EEG activity in normal elderly subjects who present abnormally high theta… with the aim of reducing the probability of posterior cognitive decline” (p. 2).

- Lines 106-108: “(…)it’s difficult to disentangle whether NF

training-related differences are due to reinforcement learning based on

contingent reward mechanisms or increased/decreased theta power levels

per se, or both.” They are not mutually exclusive. Contingency is a

condition for learning to occur. Increases or decreases in theta

activity may be a consequence of training (which always involves

contingency) or a byproduct of changes in another variable.

We agree that contingency is a condition for learning and the point we were trying to make was not clear. That is, it cannot be ruled out from previous research whether differences in Fmθ between real and sham groups are due to contingent reward mechanisms. Alternative mechanisms are also plausible (differences in motivation/engagement, attentional effort, boredom, fatigue, etc.), especially considering that sham control subjects have been shown to report knowledge of lack of contingency (Brandmeyer & Delorme, 2020). Further, in many studies the sham group also showed increases in pre-post task performance, suggesting that behavioral changes may be related to some non-specific factors unrelated to contingent reward of NF training. Thus, we argue that research is needed which consists of control and experimental groups each receiving contingent reward, but differing in the direction of targeted Fmθ modulation. In particular, previous NF research consists of training prolonged sustained Fmθ activation (5-30 min), whereas in the performance of executive tasks, frontal theta phasically increases and decreases over the course of trials. In this regard, we agree with Papo (2019) that NF training should consider dynamics-to-function mapping (although our study was designed and conducted before Papo (2019)). We have attempted to present a more clear argument in our revised Introduction, particularly under the subheading “Unresolved Issues and Gaps”.

- Lines 108-112: The concepts of contingency and learning are

inaccurate. The need that the authors mention to implement control

conditions with contingent reward is not supported by the previous ideas.

We disagree with this criticism. It is possible to disentangle the question of whether changes in Fmθ over training sessions are associated with reinforcement learning based on contingent reward mechanisms or based on differences in other potential cognitive factors such as motivation/engagement, attentional effort, boredom, fatigue, etc. We attempted to make this argument more clearly in the revised Introduction.

OVERALL PROBLEMS:

- EEG was recorded exclusively during NF training, which may not reflect

the EEG activity of the individuals at rest.

To evaluate training-related changes in Fmθ, we evaluated Fmθ relative to previous training power levels of Fmθ, except in the first session, for which we used resting Fmθ as the threshold power level.

- As the authors stated “It is a highly attention-demanding task that

must be learned through extended deliberate practice like many skills”

(lines 54-55). For this reason, 5 sessions may be insufficient for the

actual NF learning to occur.

We agree that five sessions is not sufficient extended deliberate practice. This point was made to elucidate a general issue of NF training, which is that most studies (including ours) do not train individuals over sufficient time to observe robust and lasting learning effects. We have moved this point from the Introduction to the Discussion under the sub-heading “Limitations and Future Directions”.

- Learning curves for each individual should be taken into consideration

to decide wether the participant actually learned to modify what the NF

protocol intended to modify.

Because a multi-level modeling approach was used individual variance (i.e. changes at the individual level) were accounted for. As mentioned at the end of the statistical analyses section a responder versus non-responder (i.e. whether a participant successfully altered their theta over time) analysis indicated that differences were not observed between responders and non-responders:

“A responder-only analysis was also conducted but not reported due to few differences in the results compared to using the full data. For the analysis, participants were considered responders if either session or moderation could predict their Fmθ power during NF training using univariate regression analyses. Using these criteria 18 participants were considered responders and 12 were non-responders. Again, due to limited impact on the results the full data were used”.

- The change in fmTheta may actually be due to the SH task or the rich

and novelty of the VR. The NF protocol could have nothing to do with it.

No differences in Fmθ were observed in the SH task, only in the NF training were Fmθ differences observed. Further, NF training occurred before SH in each session, except the first, so it’s unlikely novelty effects persisted across all sessions, or that the SH task in prior sessions affected the NF training in subsequent sessions.

- An adequate methodology should include, at least, 2 other control

groups: One that receives contingent reinforcement for the reduction of

fmTheta and another group that receives SHAM feedback. Only then can the

research question be truly addressed.

We agree with this statement and have included it in the section “Limitations and Future Directions”. Ideally we would have included a sham control group and a down-modulation group, however, practical considerations need to be considered as well (time, cost, subject and experimentor commitments, etc.).
