# Supplementary material for: Neural and behavioral adaptations to frontal theta neurofeedback training: A proof of concept study
Source: PLoS One. 2023 Mar 23;18(3):e0283418. doi: 10.1371/journal.pone.0283418 (PMC10035884; doi:10.1371/journal.pone.0283418)
Supplement: Attachment — Submitted filename: R1 comments.docx [file pone.0283418.s005.docx]

The reviewers and I think that the authors have improved the manuscript substantially, meanly the Introduction. However, the current version of the manuscript does not fully ready for publication yet.

Although the response letter addressed the reviewers comments, the manuscript still does not reflect what was written in the response letter. I suggest that the authors indicate precisely the lines of the document in which these changes are reflected.

The most serious problem of the article is the very small number of sessions. It is a pity that only 5 sessions have been applied, because in my opinion they are insufficient to reach a conclusion. Please review Alatorre-Cruz et al. (2022). In the penultimate paragraph of their discussion ("A strength of our study is that 30 sessions of treatment were applied...") reference is made to John Garcia's classic work on Pavlovian flavor conditioning. Even in extreme cases of survival, it is sometimes not possible to establish learning with a few training sessions. The experiment carried out in this paper is not related to survival. Therefore, although the exposed theory is interesting, it should be handled as a proof of concept without trying to reach conclusions based on the experiment. The Discussion must be much more critical regarding this weakness of the study.

Here are some suggestions that could help improve the introduction:

**Introduction**

1.- Neurofeedback (NF) is a psychophysiological training, therefore there must be scientific evidence of a clear relationship between the particular neurophysiological marker targeted by neurofeedback and the behavioral or cognitive process being studied. This is not clear in the description of NF the authors give.

2.- line 51 & 55

**"involves defining: i) the general goal;..."**

**"Depending on one’s goals, training..."**

researcher's goal regarding what? conduct? EEG? another thing?

3.- line 87-90

**"For example, Enriquez-Geppert et al., (2014) found that N-back and task-switching performance improved pre-post training in both NF and sham groups, but to a greater extent in the NF group; however, performance on stop-signal and Stroop tasks did not change as a function of real or sham training."**

If both groups improved their performance, this improvement is not an exclusive consequence of operant conditioning. What happened in the Sham group could be a consequence of other factors (see Alatorre et al 2022).

4.- lines 94-100

**"Less research has used NF to promote *decreases* in theta power. In one study using this protocol, older participants instructed to sustain decreased levels of theta power, presented as the lead where theta/alpha ratios were most abnormally high (in 6 of 14 total subjects a frontal lead was identified), the NF group exhibited higher performance gains on verbal comprehension and verbal IQ relative to a random feedback group, but no differences were observed on executive IQ (Becerra et al., 2011). Taken together, there is preliminary evidence that either up-regulating or down-regulating Fm can potentially promote gains in executive performance of some tasks."**

I invite the authors to review the article by Alatorre-Cruz et al. (2022), recently published by the same research group as Becerra et al. (2012). I consider that it is very important to highlight that the healthy older adults in both articles had an electroencephalographic risk of developing cognitive impairment (MCI or dementia); that risk consisted of having an abnormally high value of absolute power theta. This marks a big difference between the two types of participants that are pointed out: some are healthy young adults (it is not known if theta activity is normal or not) and others are clinically healthy older adults with abnormal theta activity. In the former, the objective is to improve performance but in the latter, the objective is to prevent cognitive deterioration.

The authors talk about the theta/alpha ratio, but this seems to be an error because it is not directly related to the study' rationale and is not mentioned by Alatorre-Cruz et al., who said they are replicating the Becerra experiment and evaluating a 1-year follow-up.

5.- lines 105-108

**"Considering that accurate contingent feedback linking response and reward is required for optimal learning to occur (Sterman & Egner, 2006), it’s difficult to disentangle whether NF training-related differences are due to reinforcement learning based on contingent reward mechanisms or increased/decreased theta power levels per se, or both. Contingent reward enables the accurate coupling of top-down goals and bottom-up stimulus processing (error monitoring and control) that may account for the learning differences observed in previous sham control studies. However, a need exists to implement control conditions in which accurate contingent reward is also inherent in the training and consistent with experimental NF training conditions. However, a need exists to implement control conditions in which accurate contingent reward is also inherent in the training and consistent with experimental NF training conditions."**

I exhort to the authors to better explain the ideas because I do not understand. The conclusions that are given throughout the paragraph are not derived from what has been said previously.

6.- lines 113- 117

**"Another key limitation of existing NF studies, including those targeting Fmθ, is that they modulate neural activity only in one direction (either solely up-modulation or solely down-modulation). However, to support adaptive behavior in real-world situations, neural activity must exhibit dynamic variability such that activation and inhibition must be coordinated in response to varied internal/external demands. "**

References are required

7.- lines 123-127

**" In these views, it is not sustained increases or decreases in theta (or any other neural process) that are most optimal for adaptive cognitive performance. Rather, dynamically shifting between increasing and decreasing activity (up and down modulation) might promote more efficient neural adaptations in support of superior executive control performance."**

Is this the authors' interpretation of what was previously said or are there experiments that prove it?

8.- lines 128-131

**Representing another key issue, analogous to the above on sham control issues, the Fm activation targeted by NF is often confounded with the neural functions required to modulate brain responses in NF protocols. Specifically, NF is an attention-demanding task requiring the activation of frontal executive control networks during training irrespective of the frequency or region targeted by feedback**

It is not clear "the Fm activation targeted by NF is often confounded with the neural functions required to modulate brain responses in NF protocols."

9.- line 141:

**"This possibility obscures extant findings on Fmθ modulation, such that observed effects of Fmθ modulation on the brain and performance may not be driven by NF *per se* (i.e. reinforcement learning) but instead by differences in cognitive control strategies and effort levels when attempting NF (i.e. theta activation)"**

When authors say "(i.e. reinforcement learning)" are they referring to "operant conditioning"? Terms must be used precisely. Operant conditioning can be done using reinforcers or punishments.

10.- lines 180-189

**" We tested two competing hypotheses regarding the effects of NF training on concomitant levels of Fmθ power. If Fmθ changes are due to reinforcement learning afforded by accurate contingent feedback, then the INC group should exhibit greater increases within and across training sessions. If Fmθ changes are due to greater executive task demands associated with task-switching of top-down goals, then the ALT group should exhibit greater increases within and across training sessions. Assuming that Fmθ is effectively shaped by reinforcement learning in both groups, we also hypothesized that the ALT group will improve at a greater rate on the Go-NoGo shooting task over sessions. Furthermore, we hypothesized higher Fmθ power in the ALT group during performance of the Go-NoGo task over sessions, suggesting more refined task specificity and context dependent adaptations, or more specific dynamics-to-function mapping, in the ALT group."**

Please try to explain more clearly the rationale of these hypotheses.
