# Supplementary material for: Neural and behavioral adaptations to frontal theta neurofeedback training: A proof of concept study
Source: PLoS One. 2023 Mar 23;18(3):e0283418. doi: 10.1371/journal.pone.0283418 (PMC10035884; doi:10.1371/journal.pone.0283418)
Supplement: Attachment — Submitted filename: Responses to R1 comments 2.docx [file pone.0283418.s006.docx]

The reviewers and I think that the authors have improved the manuscript substantially, meanly the Introduction. However, the current version of the manuscript does not fully ready for publication yet.

Although the response letter addressed the reviewers comments, the manuscript still does not reflect what was written in the response letter. I suggest that the authors indicate precisely the lines of the document in which these changes are reflected.

For responses to reviewers for the second revision, lines of revised text refer to the “Revised Manuscript with Track Changes” to facilitate navigation for the editor via track changes.

The most serious problem of the article is the very small number of sessions. It is a pity that only 5 sessions have been applied, because in my opinion they are insufficient to reach a conclusion. Please review Alatorre-Cruz et al. (2022). In the penultimate paragraph of their discussion ("A strength of our study is that 30 sessions of treatment were applied...") reference is made to John Garcia's classic work on Pavlovian flavor conditioning. Even in extreme cases of survival, it is sometimes not possible to establish learning with a few training sessions. The experiment carried out in this paper is not related to survival. Therefore, although the exposed theory is interesting, it should be handled as a proof of concept without trying to reach conclusions based on the experiment. The Discussion must be much more critical regarding this weakness of the study.

Thank you for these important comments and for the reference to Alatorre-Cruz et al. (2022). We agree that five sessions is very limited and insufficient to reach any strong conclusions, and that we should be more cognizant to handle the design and results as a proof of concept without trying to reach strong conclusions based on the experiment. Although our results do show statistically significant differences between groups in frontal theta power over training sessions, and differences in RT and accuracy on the Go-NoGo task in the analyses of all participants, these results lack robust statistical power. Further, we concur with the reviewers that the responders-only analyses should be reported. In more carefully reviewing the responder-only analyses, we did observe some differences in the results that we previously may have overlooked (See below under “Responders-Only Analyses”).

Accordingly, we also agree that our interpretations should be tempered and more cautiously reported, while more clearly citing limitations and highlighting proof-of-concept to better stimulate future research based on the novel concepts offered.

We have revised the Discussion to emphasize these issues (see below under “Discussion Revisions”).

Here are some suggestions that could help improve the introduction:

**Introduction**

1.- Neurofeedback (NF) is a psychophysiological training, therefore there must be scientific evidence of a clear relationship between the particular neurophysiological marker targeted by neurofeedback and the behavioral or cognitive process being studied. This is not clear in the description of NF the authors give.

We have included the following statement for clarity about clear scientific evidence of neurofeedback training effects in our description of neurofeedback training: “Scientific evidence of a clear relationship between the particular neural feature targeted by NF training and the behavioral or cognitive function being studied is measured by unambiguous changes in the targeted neural feature and by changes in the targeted behavioral or cognitive function (Rogala et al., 2016)”. [Lines 62-66]

2.- line 51 & 55 [currently line 58 & 61]

**"involves defining: i) the general goal;..."**

**"Depending on one’s goals, training..."**

researcher's goal regarding what? conduct? EEG? another thing?

We have included the following sentence to clarify the NF training goal: “In the present study, the goal is to induce changes in frontal theta power to produce changes in executive task performance”. [Lines 65-66]

3.- line 87-90

**"For example, Enriquez-Geppert et al., (2014) found that N-back and task-switching performance improved pre-post training in both NF and sham groups, but to a greater extent in the NF group; however, performance on stop-signal and Stroop tasks did not change as a function of real or sham training."**

If both groups improved their performance, this improvement is not an exclusive consequence of operant conditioning. What happened in the Sham group could be a consequence of other factors (see Alatorre et al 2022).

Please note that while this quote was found in the original submission draft, it was not included in the second version of the paper. Instead, we state: “They [Enriquez-Geppert et al., (2014)] found that the NF group exhibited higher post-test event-related (phasic) Fmθ power and performance improved pre-post training in both NF and pseudo NF groups, but to a greater extent in the NF group on two of the four tasks (three-back and task switch) vs. the pseudo NF group” [lines 122-126]

We agree with this statement and have included this point in our review of Enriquez-Geppert et al. (2014): ”The finding of performance increases in the pseudo NF group in three-back and task switch tasks suggests non-specific effects such as learning, attention, effort, etc. (Altorre-Cruz et al., 2022; Gruzelier, 2014)”. [Lines 125-127]

Related to this issue, and in light of the revisions made in the second version, we included a new sentence in our review of Wang & Hsieh (2013) to better consolidate the consistencies and inconsistencies of the findings of the more detailed reviews of Enriquez-Geppert et al. (2014) and Brandmeyer & Delorme et al. (2022): “Here again, memory function was improved with Fmθ NF training, but in contrast to Enriquez-Geppert et al. (2014b) and Brandmeyer & Delorme, (2020), conflict monitoring and attentional orienting functions also improved, suggesting generalizability of Fmθ NF training”. [Lines 158-161]

4.- lines 94-100 [current lines ]

**"Less research has used NF to promote *decreases* in theta power. In one study using this protocol, older participants instructed to sustain decreased levels of theta power, presented as the lead where theta/alpha ratios were most abnormally high (in 6 of 14 total subjects a frontal lead was identified), the NF group exhibited higher performance gains on verbal comprehension and verbal IQ relative to a random feedback group, but no differences were observed on executive IQ (Becerra et al., 2011). Taken together, there is preliminary evidence that either up-regulating or down-regulating Fm can potentially promote gains in executive performance of some tasks."**

I invite the authors to review the article by Alatorre-Cruz et al. (2022), recently published by the same research group as Becerra et al. (2012). I consider that it is very important to highlight that the healthy older adults in both articles had an electroencephalographic risk of developing cognitive impairment (MCI or dementia); that risk consisted of having an abnormally high value of absolute power theta. This marks a big difference between the two types of participants that are pointed out: some are healthy young adults (it is not known if theta activity is normal or not) and others are clinically healthy older adults with abnormal theta activity. In the former, the objective is to improve performance but in the latter, the objective is to prevent cognitive deterioration.

The authors talk about the theta/alpha ratio, but this seems to be an error because it is not directly related to the study' rationale and is not mentioned by Alatorre-Cruz et al., who said they are replicating the Becerra experiment and evaluating a 1-year follow-up.

We agree that these are important details (i.e., that older adults with abnormally high value of absolute power theta are at risk of developing cognitive impairment versus young or healthy older participants for performance enhancement. We have included these points in our review of Becerra et al. (2012) and included Alatorre-Cruz et al. (2022) [line 165]:

“Further, it should be noted that NF training goals for clinically healthy older adults with abnormally high levels of theta activity are to prevent cognitive deterioration more so than to improve cognitive performance as is the goal for healthy participants”. [lines 176-179]

Regarding theta/alpha ratios, we state that “Often these studies also involve simultaneous modulations of other frequency bands (e.g., alpha, sensorimotor rhythm, beta) and brain regions (e.g., central, parietal) so the specific role of Fmθ in NF training is not always clear”, which refers to the collection of citations “(Alatorre-Cruz et al., 2022; Arnold et al., 2021; Becerra et al., 2011; Campos da Paz et al., 2018; Van Doren et al., 2018)” and is not specifically referring to Becerra et al. (2012) or Alatorre-Cruz et al. (2022).

5.- lines 105-108

**"Considering that accurate contingent feedback linking response and reward is required for optimal learning to occur (Sterman & Egner, 2006), it’s difficult to disentangle whether NF training-related differences are due to reinforcement learning based on contingent reward mechanisms or increased/decreased theta power levels per se, or both. Contingent reward enables the accurate coupling of top-down goals and bottom-up stimulus processing (error monitoring and control) that may account for the learning differences observed in previous sham control studies. However, a need exists to implement control conditions in which accurate contingent reward is also inherent in the training and consistent with experimental NF training conditions. However, a need exists to implement control conditions in which accurate contingent reward is also inherent in the training and consistent with experimental NF training conditions."**

I exhort to the authors to better explain the ideas because I do not understand. The conclusions that are given throughout the paragraph are not derived from what has been said previously.

It appears this quote was taken from the first version of the paper. We cannot find this specific quote in the second version of the paper. In the second version, we attempted to clarify our ideas by citing potential alternative mechanisms which may underlie findings in previous Fmθ NF studies using sham control groups which requires further inquiry and hypothesis testing:

“Considering that accurate contingent feedback linking response and reward is required for optimal learning to occur (Sterman & Egner, 2006), it’s assumed that participants receiving real NF training have learned to modulate specific EEG features (e.g., Fmθ), while sham participants have not. However, **it cannot be ruled out from previous research whether differences in Fmθ between real and sham groups are due to self-regulation based on contingent reward learning mechanisms.** **Alternative mechanisms are also plausible**, e.g., increased frontal theta has also been shown to be associated with attentional effort/task demand (Gevins et al., 1997; Rajan et al., 2018), decision making (Womelsdorf, Vinck, Leung, & Everling, 2010), working memory (Jensen & Tesce, 2002), and boredom/fatigue (Tran et al., 2020; Wascher et al., 2014), to list a few. Thus, **it’s conceivable that differences in Fmθ may also be at least partially attributable to other cognitive functions**, especially considering that sham control participants have been shown to report knowledge of lack of contingency between their strategies and feedback signals (Brandmeyer & Delorme, 2020), which may reduce motivation and attentional engagement. It’s also common that subsets of NF training participants are non-responders (Brandmeyer & Delorme, 2020; Enriquez-Geppert et al., 2014b), revealing that even with accurate contingent feedback some participants are unable to increase Fmθ. Further, **sham groups have also shown increases in pre-post task performance (Enriquez-Geppert et al., 2014a), suggesting that behavioral changes may be related to some non-specific factors unrelated to contingent reward mechanisms of NF training**. Contingent reward enables the potential for accurate coupling of top-down goals and bottom-up stimulus processing (error monitoring and control) that may account for the learning differences observed in previous sham control studies. However, **a need exists to compare NF training protocols in which accurate contingent reward is held constant, while manipulating other parameters of training, such as the top-down training goals (e.g., increase versus decrease Fmθ)**”. [Lines 195-216]

6.- lines 113- 117 [current lines 217-221]

**"Another key limitation of existing NF studies, including those targeting Fmθ, is that they modulate neural activity only in one direction (either solely up-modulation or solely down-modulation). However, to support adaptive behavior in real-world situations, neural activity must exhibit dynamic variability such that activation and inhibition must be coordinated in response to varied internal/external demands. "**

References are required

The sentence immediately following the above quoted text provides numerous references citing these concepts:

“There are a number of theoretical concepts rooted in nonlinear systems that underscore the importance of such dynamic variability such as self-organized criticality (Deco, Jirsa, & McIntosh, 2013; Lewis, 2005; Werner, 2007; Van Orden, Holden, & Turvey, 2003; Mahmoodi, West, & Grigolini, 2017), multistability (Fingelkurts, Fingelkurts, & Neves, 2010; Tognoli & Kelso, 2014; Werner, 2013), and coordination dynamics (Tognoli & Kelso, 2014; Werner, 2007). Here, a system (e.g., the brain) with high levels of dynamic variability is on the verge of instability, such that this system is more flexible (i.e., neural resources can be more efficiently adapted to changing internal and external demands)”. [Lines 221-227]

7.- lines 123-127 [current lines 227-231]

**" In these views, it is not sustained increases or decreases in theta (or any other neural process) that are most optimal for adaptive cognitive performance. Rather, dynamically shifting between increasing and decreasing activity (up and down modulation) might promote more efficient neural adaptations in support of superior executive control performance."**

Is this the authors' interpretation of what was previously said or are there experiments that prove it?

These statements provide the theoretical bases for the research hypotheses and study design based on theoretical models and empirical results of EEG research from a complex systems perspective cited immediately prior:

“Another key limitation of previous NF studies, including those targeting Fmθ, is that they modulate neural activity only in one direction (either solely up-modulation or solely down-modulation). However, to support adaptive behavior in real-world situations, neural activity must exhibit dynamic variability such that activation and inhibition must be coordinated in response to varied internal/external demands. There are a number of theoretical concepts rooted in nonlinear systems that underscore the importance of such dynamic variability such as self-organized criticality (Deco, Jirsa, & McIntosh, 2013; Lewis, 2005; Werner, 2007; Van Orden, Holden, & Turvey, 2003; Mahmoodi, West, & Grigolini, 2017), multistability (Fingelkurts, Fingelkurts, & Neves, 2010; Tognoli & Kelso, 2014; Werner, 2013), and coordination dynamics (Tognoli & Kelso, 2014; Werner, 2007). Here, a system (e.g., the brain) with high levels of dynamic variability is on the verge of instability, such that this system is more flexible (i.e., neural resources can be more efficiently adapted to changing internal and external demands)”. [Lines 217-227]

To our knowledge, there are no neurofeedback studies designed or tested from this theoretical perspective, although Papo (2019) provides strong arguments and recommendations for doing so, which we state on lines 233-234: “In this regard, we agree with Papo (2019) that NF training should consider dynamics-to-function mapping”.

8.- lines 128-131 [current lines 239-242]

**Representing another key issue, analogous to the above on sham control issues, the Fm activation targeted by NF is often confounded with the neural functions required to modulate brain responses in NF protocols. Specifically, NF is an attention-demanding task requiring the activation of frontal executive control networks during training irrespective of the frequency or region targeted by feedback**

It is not clear "the Fm activation targeted by NF is often confounded with the neural functions required to modulate brain responses in NF protocols."

Here we were attempting to state that frontal theta increases in association with executive task demands, and that NF training, regardless of the targeted frequency band, brain region, or real or sham conditions, imposes executive task demands, thereby increases in frontal theta activity. To make this more clear, we have included reference to the above comment by Gruzelier (2014):

“For example, Gruzelier (2014) contended that “evidence that a frontal locus is the strongest locus showing changes following training, though this is not an exclusive locus. Even posterior training predominantly leads to stronger frontal than posterior effects” (p. 167) and that Fmθ “may represent a nexus of factors including learning, self-regulation, as well as non-specific issues such as attention and effort, but perhaps most central of all action monitoring” (p. 171). Because Fmθ is implicated in such a nexus of executive control processes, observed Fmθ increases during NF training may be erroneously attributed to effective NF training (i.e. self-regulation based on accurate contingent feedback) when such increases may merely indicate sustained deployment of attention, effort, and action monitoring”. [Lines 243-251]

9.- line 141: [current lines 256-260]

**"This possibility obscures extant findings on Fmθ modulation, such that observed effects of Fmθ modulation on the brain and performance may not be driven by NF *per se* (i.e. reinforcement learning) but instead by differences in cognitive control strategies and effort levels when attempting NF (i.e. theta activation)"**

When authors say "(i.e. reinforcement learning)" are they referring to "operant conditioning"? Terms must be used precisely. Operant conditioning can be done using reinforcers or punishments.

Given the revisions in response to comment 8 above, we also modified the quoted statement in comment 9 to:

“This possibility obscures extant findings on Fmθ modulation, such that observed effects of Fmθ modulation on the brain and performance may not be driven by NF *per se* (i.e. self-regulation) but instead by differences in theta activation related to attention engagement or other non-specific executive control processes when attempting NF”. [Lines 256-260]

We have adopted the term “self-regulation” throughout the rest of the paragraph, and the following paragraph, in place of “reinforcement learning” for consistency and clarity. [Lines 261, 269, 272, 273, 298, 304, 308]

We also incorporated use of the term self-regulation throughout the Discussion.

10.- lines 180-189 [current lines 302-316]

**" We tested two competing hypotheses regarding the effects of NF training on concomitant levels of Fmθ power. If Fmθ changes are due to reinforcement learning afforded by accurate contingent feedback, then the INC group should exhibit greater increases within and across training sessions. If Fmθ changes are due to greater executive task demands associated with task-switching of top-down goals, then the ALT group should exhibit greater increases within and across training sessions. Assuming that Fmθ is effectively shaped by reinforcement learning in both groups, we also hypothesized that the ALT group will improve at a greater rate on the Go-NoGo shooting task over sessions. Furthermore, we hypothesized higher Fmθ power in the ALT group during performance of the Go-NoGo task over sessions, suggesting more refined task specificity and context dependent adaptations, or more specific dynamics-to-function mapping, in the ALT group."**

Please try to explain more clearly the rationale of these hypotheses.

To be more consistent and clear with respect to the changes made above (responses to comments 8 & 9), we have revised our rationale of our hypotheses:

“We tested two competing hypotheses regarding the effects of NF training on concomitant levels of Fmθ power. If Fmθ changes are due to self-regulation afforded by accurate contingent feedback, then the INC group should exhibit greater increases within and across training sessions. If, on the other hand Fmθ increases more in the ALT group, or no differences are observed between groups, then self-regulation based on accurate contingent reward mechanisms underlying reinforcement learning would be challenged. Assuming that Fmθ is effectively shaped by self-regulation in both groups, we hypothesized that the INC group would exhibit greater increases in Fmθ within and across NF training sessions. However, in contrast with previous research, we hypothesized that the ALT group would improve at a greater rate on the Go-NoGo shooting task over sessions, implying that training to dynamically modulate increases and decreases in Fmθ provides greater training specificity of dynamics-to-function mapping than does training to systematically increase Fmθ. Furthermore, we hypothesized higher Fmθ power in the ALT group during performance of the Go-NoGo task over sessions, suggesting more refined task specificity and context-dependent adaptations, or more specific dynamics-to-function mapping, in the ALT group”. [Lines 302-316]

Also, to better set up the rationale, we also revised the preceding paragraph:

“Here, up/down modulation would require alternating top-down NF goals. As such, if up/down-modulation promotes lower Fmθ levels, particularly in blocks of down- versus up-modulation within the alternating group, and relative to the group trained in up-modulation alone, then NF training adaptations are likely due to self-regulation based on accurate contingent feedback. If, on the other hand, up/down modulation promotes higher Fmθ levels relative to up-modulation alone, then NF training adaptations are likely due to some other mechanism unrelated to self-regulation. In other words, if observed Fmθ increases are in fact due to NF training effects (self-regulation), then up-modulation should promote greater Fmθ than alternating up and down modulation. Further, if training-related Fmθ power increases are associated with improvements in executive task performance, then up-modulation training should lead to superior executive task performance”. [Lines 263-276]

Reviewer #3: The authors have addressed in an adequate manner the reviewers comments in their response letter. The aim of the study seems more clear to me now.

However, the manuscript still does not reflect what was written in the response letter to the reviewers. The issue of mereological fallacy is still found throughout the manuscript (e.g. lines 67 & 70).

The criticism that “mereological fallacy is still found throughout the manuscript” is not clear to us. To the extent we can address this comment, we agree that part-whole relationships are difficult to disentangle and such ideas come from reductionistic approaches in science. In our view, based on complex systems (holistic approaches), the properties of the system are distinct from the properties of the parts, and they depend on the properties of the whole. However, it’s not clear to us how we bring these ideas into the current paper, especially considering the vast literature on NF training, which assumes associations between specific brain processes and specific cognitive/behavioral processes, although it is understood that there is no one-to-one mapping between the two. We believe our review of the literature in the Introduction highlights non-specific effects and that Fmθ is associated with a nexus of executive control [lines 246-248] and other cognitive processes: “attentional effort/task demand (Gevins et al., 1997; Rajan et al., 2018), decision making (Womelsdorf, Vinck, Leung, & Everling, 2010), working memory (Jensen & Tesce, 2002), and boredom/fatigue (Tran et al., 2020; Wascher et al., 2014), to list a few” [lines 201-203]

Please make sure that the whole manuscript is coherent with the answers that you have provided to the reviewers.

Moreover, I encourage the author to include the results of the comparison between responders and non-responders. Even though you have found no differences, this is an important issue, because it is directñy related to your research question.

Responder-Only Analyses

Based on reviewer comments, we have looked more carefully at our responder-only analyses and some of the results are indeed different. We agree that providing these results will benefit the paper. However, because doing so would require much more space to an already lengthy paper, we propose to present these results in supplementary materials. In the supplementary materials, we present statistical tables and figures for (1) Fmθ changes over blocks and sessions of NF training for each group, (2) Fmθ changes over sessions in low and high time-stress conditions of Go-NoGo shooting task for each group, and (3) performance changes (errors of commission, shooting accuracy, and reaction times) over sessions in low and high time-stress conditions of Go-NoGo shooting task for each group (with the same structure and format as presented for the main results in the manuscript).

Perhaps the most important finding from these analyses, is the emergence of a main effect for Group in the analysis of Fmθ changes over blocks and sessions of NF training, despite significant interaction effects as in the analyses of all participants (Group x Block, Group x Session, and Group x Session x Block). Specifically, the INC group exhibited higher Fmθ than the ALT group, which is a meaningful result that must be integrated in revision of the Discussion.

No differences were observed in the analyses of Fmθ changes over sessions in low and high time-stress conditions of Go-NoGo shooting task for each group, as in the analyses of all participants.

For the analyses of performance changes (errors of commission, shooting accuracy, and reaction times) over sessions in low and high time-stress conditions of Go-NoGo shooting task for each group, the following differences were observed:

1) Errors of commission: Condition and Session main effects were significant in the analyses of all participants, but were no longer significant in the responders-only analyses
2) Accuracy: The Group x Session interaction was significant in the analyses of all participants, but was no longer significant in the responders-only analyses
3) Reaction Times: The Condition x Session and the Group x Condition interactions were significant in the analyses of all participants, but were no longer significant in the responders-only analyses

In light of these findings, we have revised the “Statistical Analyses” section. We have created a new subheading, “Responder-Only Analyses” and revised the text to refer to the supplementary materials section, and remove text stating limited impact:

Responder-Only Analyses

“Responder-only analyses were also conducted, which are reported in supplementary materials. For the analyses, participants were considered responders if either session or moderation could predict their Fmθ power during NF training using univariate regression analyses. Using these criteria 18 participants were considered responders and 12 were non-responders”. [Lines 608-614]

We also include the above text in the supplementary sect.

Discussion Revisions

In response to the comment “The Discussion must be much more critical regarding this weakness [limited number of training sessions] of the study”, we have made the following revisions:

Added: “Overall, the present findings indicate greater Fmθ increases across training sessions in the ALT versus INC group. Accuracy and reaction time metrics improved at a steeper rate in the INC relative to the ALT group. Taken together, these results are consistent with the notion that alternating up- and down-modulation of Fm incurred switching costs that led to overall increases in theta (across sessions). It should be noted that these inferences are merely suggestive in light of insufficient number of sessions to explore alternative accounts of the findings (e.g., learning). Future studies should corroborate these findings using more practice sessions. Nevertheless, this study is an important for step in unraveling the breadth of complex neurocognitive mechanisms at play in NF protocols.” [lines 728-736]

Added: “However, these interpretations must be tempered based on one difference observed in the responder-only analysis (supplementary materials). That is, a main effect of Group was observed such that participants in the INC group exhibited higher Fm than the ALT group. These findings must also be interpreted with caution because of the loss of statistical power when analyses are conducted on a subset of the data (n=18). Also, considering the relatively small number of training sessions, it is unlikely that sufficient training was provided in the present study.” [lines 804-810]

Deleted: “Our analysis of responders-only (n=18), using univariate regression analyses to determine if either session or moderation could predict Fm power during NF training, revealed comparable results. This finding indicates that, even when non-responders were removed, responders in the ALT group still exhibited greater Fmθ increases within and across sessions.” [lines 836-839]

Added: “However, in the responder-only analysis, this effect was non-significant” [lines 852-853]

Added: “However, in the responders-only analysis, interaction effects were non-significant.” [lines 859-860]

Added: “Our results are difficult to form any firm conclusions given its limitations. However, we hope that we have introduced a proof-of-concept based on new ideas to better stimulate future research based on the novel concepts offered.” Lines 969-971]

Deleted: “suggest that training-related Fmθ changes were not explained by reinforcement learning afforded by accurate contingent feedback, but rather by differences in NF training demands, perhaps imposed by reactivation of contextual task rule representations related to consistent (INC) versus alternating (ALT) top-down training goals.” [lines 971-974]

Added: “Finally, more training sessions should be provided, and follow-up studies are needed to better understand longer-term effects of NF training (Altorre-Cruz et al., 2022).” [lines 993-995]
